# Psychometric data on knowledge and fear of coronavirus disease 2019 and perceived stress among workers of filipino origin in Hong Kong

**DOI:** 10.1016/j.dib.2020.106395

**Published:** 2020-10-09

**Authors:** David Chun Yin Li, Ling Leung

**Affiliations:** Department of Surgery, University of Hong Kong, Hong Kong Special Administrative Region, Hong Kong

**Keywords:** COVID-19, Health knowledge, Fear perceptions, Work Stress

## Abstract

Data on knowledge and fear of coronavirus disease 2019 (COVID-19) and perceived stress were collected in July 2020 from a convenience sample of Filipino domestic workers in Hong Kong by asking participants to take part in three questionnaires. First, twelve questions related to knowledge associated with the prevention and identification of COVID-19 were used to assess participants’ knowledge regarding COVID-19. Second, the Fear of COVID-19 Scale (FCV-19S) was used to assess participants’ perceived fear of infection. Third, the Short Form Perceived Stress Scale (PSS-4) was used to measure participants’ perceived stress. Pearson product-moment correlation coefficients were obtained to assess the relationships between the total scores of the three questionnaires. The relationship between knowledge of COVID and fear of COVID was significant, *r(108) = +0.23, p = .02*; the relationship between fear of COVID and perceived stress was not statistically significant, *r(108) = +0.17, p = .08*; the relationship between knowledge of COVID and perceived stress was not statistically significant, *r(108) = -0.11, p = .26*. Cronbach's alpha coefficients were obtained for each of the three questionnaires to assess internal consistency reliability.

## Specifications Table

**Table:** 

Subject	Psychology
Specific subject area	Health Psychology, Human Resource Management Public, Health and Health Policy
Type of data	Tables
How data were acquired	Convenience sampling
Data format	Raw, Analyzed
Parameters for data collection	Data were obtained from 110 respondents who self-identified as domestic helpers of Filipino origin currently working in Hong Kong. The data were collected on 18th July 2020.
Description of data collection	Non-random convenience sampling and street-intercept survey techniques were used to recruit participants.
Data source location	Central, Hong Kong Island. *22.2800° N, 114.1588° E.*
Data accessibility	Repository name: Open Science Framework (OSF) Data identification number: DOI 10.17605/OSF.IO/M2V3H Direct URL to data: https://osf.io/m2v3h/?view_only=9e48fc707cef437bb3e51510abb959aa

## Value of the Data

• This dataset is important because it provides knowledge and fear of COVID and perceived stress amongst migrant workers living in Hong Kong, an important migrant group in the local territory's communities.
• Because the effectiveness of COVID-19 preventive measures is affected by the collective knowledge and attitude of all residents, including those of ethnic minorities who are marginalized by mainstream society, health policymakers would benefit from this dataset in the context of the ongoing COVID-19 global pandemic.
• This dataset could be compared to similar surveys for different geographical regions or across different demographic groups.
• This dataset could be used for further statistical analyses or metanalyses, notwithstanding the limitation of its relatively small sample size.

## 1 Data Description

This dataset provided information on COVID-19-related knowledge, fear of COVID-19, and perceived stress among Filipino domestic helpers living in Hong Kong. Sociodemographic information about each respondent's age group and self-identified gender was collected. Each respondent was invited to participate in three questionnaires. The first questionnaire assessed respondents’ COVID-19 knowledge. The second questionnaire was the Fear of COVID-19 Scale (FCV-19S) and assessed participants’ fear of COVID-19. The third questionnaire was the Short Form Perceived Stress Scale (PSS-4) and assessed the participants’ perceived stress in the last month.

The demographic characteristics of respondents were presented in Table 1. The means and standard deviations of the three abovementioned questionnaires were presented in Table 2. Pearson bivariate correlations on the total scores of the three questionnaires were presented in Table 3. Internal consistency reliabilities of the three questionnaires were found to be acceptable by obtaining the Cronbach's alpha coefficients: *α = 0.72* for COVID knowledge, *α = 0.83* for FCV-19S, and *α = 0.89* for PSS-4. All data were uploaded onto Open Science Framework (OSF) data depository in a Microsoft Excel Binary Workbook File (.xlsb).

**Table 1:** Sociodemographic characteristics of respondents in the sample (*N* *=* *110*).

Variable	Category	Frequency	Percentage
Age	18–24	4	4%
	25–30	23	21%
	31–36	63	57%
	37–42	14	13%
	42+	6	5%

Gender	Female	108	98%
	Male	0	0%
	Other	2	2%

**Table 2:** Descriptive statistics on the questionnaires.

Variable	N	M	SD	Variance
COVID Knowledge Total Score	110	10.70	1.71	2.91
FCV-19S Total Score	110	11.81	2.97	8.82
PSS-4 Total Score	110	10.67	3.24	10.48

**Table 3:** Results of Pearson Bivariate correlation on the scores of the three questionnaires.

Variable		1	2	3
1. COVID Knowledge Total Score	Pearson Correlation	1.00		
	Sig. (2-tailed)			
	N	110		

2. FCV-19S Total Score	Pearson Correlation	0.23*	1.00	
	Sig. (2-tailed)	0.02		
	N	110	110	

3. PSS-4 Total Score	Pearson Correlation	−0.11	0.17	1.00
	Sig. (2-tailed)	0.26	0.08	
	N	110	110	110

⁎ Correlation is significant at the 0.05 level (two-tailed).

## 2 Experimental Design, Materials and Methods

This research adopted a descriptive and self-reported survey design to evaluate knowledge and fear of COVID-19 and perceived stress amongst Filipino domestic helpers living in Hong Kong. A total of 110 responses was received. The cross-sectional data were collected in Central, Hong Kong Island, on 18th July 2020, during a time of an ongoing global COVID-19 pandemic [1], [2], [3]. The data collectors used a street interceptive method to invite participants to take part by asking them to fill in paper questionnaires. A street-intercept survey method had the advantage of allowing the data collectors to screen potential participants for suitability [4,5].

The criterion for selection was an agreement with the statement that “I consider myself to be a domestic helper currently working in Hong Kong, and I am originally from the Philippines.” A total of 150 persons were approached in a non-probabilistic manner by the data collectors based on the physical appearances of the passers-by, and *N* *=* *110* responses were received. Central, a business district in Hong Kong Island measuring 0.75 square kilometers in size, was chosen as the location for the survey. This was because the public areas of Central were the primary places where Philipino workers congregate on Saturdays and Sundays. All participation was voluntary and strictly anonymous, and written consent was received from each participant.

The first questionnaire consisted of twelve items/ true or false questions adapted from the COVID's knowledge questionnaire developed by Zhong et al. [6] and assessed respondents’ COVID-19 knowledge. The second questionnaire was the FCV-19S Scale [7,8] and consisted of seven items. The third questionnaire was the PSS-4 [9,10] and consisted of four items. Both the FCV-19S and PSS-4 used a five-point Likert scale. Pearson bivariate correlations were used to assess the relationships between knowledge of COVID, fear of COVID, and perceived stress. Cronbach's alpha coefficients were obtained to assess the three questionnaires’ reliabilities [11]. All statistical analyses were carried out using IBM Statistical Package for Social Sciences Version 23.0 [12].

## Ethics Statement

The research was conducted in accordance with the Declaration of Helsinki and its subsequent amendments. Participation was entirely voluntary, anonymous, and consensual. No financial incentives were offered or provided for participation. Ethical approval was obtained from the University of Hong Kong.

## Declaration of Competing Interest

This research did not receive any specific grant from funding agencies in the public, commercial, or not-for-profit sectors. The authors declared that they had no known competing financial interests or personal relationships which have, or could be perceived to have, influenced the work reported in this dataset paper.

## Appendix Supplementary materials

Image, application 1
